# A Comparative Study of the Effect of Different Carbon-Reduction Policies on Outsourcing Remanufacturing

**DOI:** 10.3390/ijerph19063590

**Published:** 2022-03-17

**Authors:** Qiuyue Li, Hao Wang, Zhenshan Li, Shangwei Yuan

**Affiliations:** 1 College of Business Administration, Guangxi University, Nanning 530004, China; lqy0623l@163.com; 2 Department of Geography and Planning, University of Toronto, Toronto, ON M5S 2E8, Canada; haow.wang@mail.utoronto.ca; 3 School of Business, Zhengzhou University, Zhengzhou 450001, China; 4 Faculty of Social Sciences, University of Southampton, Southampton SO17 1BJ, UK; sy4u21@soton.ac.uk

**Keywords:** carbon-emission-constraint, carbon-trade, outsourcing remanufacturing, supply chain system

## Abstract

To facilitate the green transformation of enterprises and realize low-carbon development, governments have adopted the policies of carbon emission constraint and carbon trade to promote enterprises’ low-carbon production. Although the two policies aim to reduce carbon emissions, they have different effects on enterprises’ production. Meanwhile, the development of remanufacturing caters to the low-carbon economy. Therefore, this article establishes the game models between an original equipment manufacturer (OEM) and a remanufacturer under carbon-emission-constraint and carbon-trade policies, analyzing the production decisions of enterprises under different policies to compare the influence of the two policies on outsourcing remanufacturing. The main conclusions of the article are as follows: (1) Both carbon-emission-constraint and carbon-trade policies increase the unit retail price of remanufactured and new products, reducing the new products sales volume. However, the sales volume of remanufactured products only decreases if the discount rate is less than the rate of carbon emissions of the two products. (2) The upper limit of carbon emissions can affect the unit outsourcing cost. The unit cost of outsourcing under the carbon-emission-constraint policy is only higher when the upper limit of carbon emissions is less than a certain threshold, and the discount rate is larger than the proportion of carbon emissions for both products; otherwise, the unit outsourcing cost under the carbon-trade policy is higher. (3) Both policies lessen the total environmental implication. When the upper limit of carbon emissions is less than a particular threshold, the environmental effect of the two manufacturers under the carbon-emission-constraint policy is smaller; otherwise, the environmental impact is smaller under the carbon-trade policy.

## 1. Introduction

Low carbon use development is a global trend in the current international environment, and the new field of international competition is inclined towards low-carbon technology and industrial competition [1,2,3]. Due to the COVID-19 outbreak, the significant reduction in global economic activity resulted in a dramatic decrease in carbon emissions in 2020. However, the global carbon emissions rebounded to near the pre-COVID-19 levels in 2021 as many countries gradually controlled COVID-19 with the advent of COVID-19 vaccines [4]. At present, the global carbon emission situation remains grim, but the low-carbon economy is the current trend, with some major countries going through the transition stage. The U.S. has been improving its carbon-emission-reduction system since the 1970s, China has continuously promoted energy restructuring to achieve its dual carbon targets, and Europe has promoted green transformation by establishing a policy framework and a system of a carbon-emission-reduction market. To date, more than 50 countries have peaked in carbon dioxide emission, and approximately 110 countries have set the target of achieving carbon neutrality by the mid-21st century. The promotion of the green and low-carbon economy cannot be achieved without the support of policies, leading the government to implement various policies to intervene in the production of the OEM and the remanufacturer, such as carbon-emission-constraint [5,6,7,8,9,10], carbon trade [11,12,13,14,15,16,17], carbon tax [18,19,20,21,22,23,24,25], government subsidy [26,27], and so on. Among them, the market-oriented policies of carbon, also known as the carbon-emission-constraint and the carbon-trade policies, are applied by the government to restrain the excessive carbon emission of enterprises, which are the mainstream policies to realize the low-carbon economy [28,29]. Additionally, both set a carbon emission cap on the carbon emission of enterprises’ output. Under the carbon-emission-constraint policy, the carbon emissions produced by manufacturers should be below the upper limit defined by the government, or they face punitive actions by the government. Additionally, under the carbon-trade policy, if the carbon emissions generated by the manufacturer are over the upper limit set by the government, they can continue their production by purchasing additional carbon emission rights. Since the carbon-emission-constraint policy directly restricts the excessive carbon emission of enterprises by putting a cap on carbon emissions, and the transaction cost is relatively low under the carbon-trade policy, the two policies are more effective in carbon emission reduction compared with other policies [30,31].

Compared with other low-carbon production activities, the recycling and remanufacturing of used products have become a typical model that could promote a circular economy, energy conservation, and emission reduction [32,33]. Remanufacturers make not only full use of resources by recycling and remanufacturing waste products but also reduce environmental pollution in the production process [34]. However, in remanufacturing activities, the OEM lacks the advanced technology and equipment required for remanufacturing production, resulting in lower efficiency and lower profitability in the production of remanufactured products. The OEM will decide to outsource the recycling and remanufacturing process to a remanufacturer with professional technology and equipment to maximize the use of resources, yet both the new products generated by the OEM and the remanufactured products generated by the remanufacturer will be sold by the OEM, thus constituting an outsourcing remanufacturing production mode. In the outsourcing remanufacturing production model, the OEM generates new products, and the remanufacturer recovers remanufacturing production activities by charging an outsourcing fee per unit of remanufactured product, with the pricing and sales of new and remanufactured products being up to the OEM. Therefore, in the background of the application of carbon-emission-reduction policies, based on the outsourcing remanufacturing production pattern to establish a closed-loop supply chain composed of an OEM and a remanufacturer, the two manufacturers can adjust their production decisions promptly according to the policies of carbon-emission-constraint and carbon-trade. Not only can this optimize the production structure of remanufactured and new products, but it can also efficiently decrease the carbon emissions produced by the output of the manufactured products, sustainably promoting green and low-carbon manufacturing.

Against the background of green and low-carbon movements, numerous academics have researched and discussed various policies issued by the government. Scholars explored the impact of carbon-emission-reduction policies on production decisions, including corporate financing decisions under the carbon-emission-constraint policy [35,36,37], the optimal emission reduction mode of the supply chain under carbon-emission-reduction policies [38,39], ordering strategy and inventory management [7,40,41,42], and the optimal production tactic for manufacturers under the carbon-trade policy [17,43,44]. Many scholars analyzed product pricing strategies considering the effect of carbon-emission-reduction policies on product costs and prices [45,46]. Other scholars combined carbon-emission-reduction policies with recycling for analysis, providing inspiration for policy-making and remanufacturing decisions [47,48]. There are also studies that included carbon-emission-reduction policies into decision-making factors and analyzed the remanufacturing mode under the interaction of multiple factors [49,50,51], and other scholars discussed the differences among different kinds of policies [52,53,54,55,56]. Carbon-emission-reduction policies have attracted some attention. Generally, scholars studied the influence of policies on the decision-making process of enterprises. However, there is little comparative analysis on the implication of various policies on outsourcing remanufacturing. Based on this, comparing the advantages and disadvantages of the carbon-emission-constraint and carbon-trade policies, which have more obvious effects on carbon emission reduction, deserves further discussion. For example, are the production decisions of the OEM and the remanufacturer under the carbon-trade policy better than those under the carbon-emission-constraint policy? Is the carbon-trade policy better for the environment? Consequently, it is essential to conduct a comparative analysis about the impact of different policies on outsourcing remanufacturing. This article focuses on which policy is more beneficial for outsourcing remanufacturing: carbon emission constraint or carbon trade. This article analyzes the impact of the carbon-emission-constraint and carbon-trade policies on outsourcing remanufacturing production by constructing a model under different carbon-reduction policies and comparing the decision factors of carbon-reduction policies through the model.

This article focuses on outsourcing remanufacturing, a typical remanufacturing model, comparing and analyzing the effects of carbon-emission-constraint and carbon-trade policies on remanufacturing production. This article focuses on answering the following three questions:

1. What are the influential mechanisms of carbon-emission-constraint and carbon-trade policies in remanufacturing?
2. What is the impact of carbon emission constraint and carbon trade on production decisions, such as the unit retail price, sales volume, environment, etc.?
3. How can the government formulate appropriate carbon-emission-reduction policies, and which policy is more conducive to remanufacturing?

There are five sections in this paper. Section 2 outlines the problem description and game model. Section 3 describes model construction, analysis, and conclusions. Section 4 cites a case of a Chinese enterprise for numerical analysis. Section 5 draws findings and management enlightenment.

## 2. Model Introduction

### 2.1. Problem Description

Based on outsourcing remanufacturing, this article constructs a manufacturing/remanufacturing supply chain system consisting of an OEM and a remanufacturer. It presents a comparative study of the impact of carbon-emission-constraint and carbon-trade policies on the supply chain. The government can implement two policies to influence the carbon emissions of enterprises in different ways to limit carbon emissions and achieve low-carbon development. One is the carbon-emission-constraint policy, which limits the total carbon emissions by setting an upper limit of carbon emissions. Namely, companies are penalized by the government if they exceed the upper limit. The other is the carbon-trade policy, which turns carbon emission into a valuable commodity by establishing a carbon trading market. This is achieved by increasing the production cost of enterprises with excessive carbon emissions and the revenue of low-carbon enterprises. Although the two policies have different impact mechanisms, they are both disadvantageous to OEMs with high carbon emissions, so the OEM has to make decisions according to various government policies. In the outsourcing remanufacturing mode, remanufactured and new products are competing with each other, and both are sold by the OEM, which is a decision of a single oligopoly enterprise. The OEM should consider the changes in its own production cost and revenue, then decide the unit retail price and sales volume of two products. Based on outsourcing remanufacturing, this article studies the impact mechanisms of the carbon-emission-constraint and carbon-trade policies. Further, it discusses the production decisions of OEM under the two different policies.

### 2.2. Model Symbol

Table 1 displays the fundamental definitions of the notation used in this article.

### 2.3. Model Function

According to [14,57,58,59], the demand function and recovery function are as follows:

(1) Model demand function:

The demand function used in this study is a relatively mature function that was used in the domestic and international literature. This article can determine the market demand function for remanufactured and new products based on the consumer utility function, and based on [59], the perceived value of the consumer for the new product is σ, and σ obeys a uniform distribution on [0,1]. The consumer’s perceived value of the new product is δσ. The consumer’s utility from a unit of the new product is $U_{in}=\delta -p_{in}$, and the utility from a unit of the remanufactured product is $U_{ir}=\delta \theta -p_{ir}$. Consumers prefer to purchase new products when their perceived value is consistent with $U_{in}>U_{ir}$ and $U_{in}>0$, and prefer to purchase remanufactured products when their perceived value is consistent with $U_{ir}>U_{in}$ and $U_{ir}>0$. Since σ obeys a uniform distribution of [0,1], the market demand for the two products can be obtained after integration as $q_{in}=\frac{1-\delta -p_{in}+p_{ir}}{1-\delta },q_{ir}=\frac{\delta p_{in}-p_{ir}}{\delta (1-\delta )}$, respectively; the inverse demand function of consumers for the two products can be expressed as, where i∈{CE,CO,CD}. Additionally, the new product output should meet $q_{in}<\frac{1}{\tau_{i}+1},q_{ir}<\frac{\tau_{i}}{\tau_{i}+1}$; otherwise, the retail price of the new and remanufactured products will likely have negative values, which is not in line with reality.

(2) Model recovery function:

The remanufacturer needs to recycle the new products sold by the OEM when carrying out remanufacturing production activities, so the recycling cost is included in the remanufacturing cost. By drawing on the literature [14], it can be seen that the recycling cost is positively related to the recycling quantity and is a convex function of the recycling quantity, and the recycling function can be expressed as $\frac{k}{2}{\left(\tau_{i}q_{in}\right)}^{2}$, where $i\in \left\{CE,CO,CD\right\}$, k denotes the recycling coefficient of the used product.

### 2.4. Research Hypothesis

**Hypothesis** **1.** *In the outsourcing remanufacturing production model, the OEM only produces new products, the OEM outsources remanufacturing activities to a remanufacturer through outsourcing behavior, and the OEM pays the remanufacturer the total outsourcing cost based on the outsourcing cost per remanufactured product and the volume of remanufactured products. The OEM can influence the remanufacturer’s production decisions by changing the outsourcing cost per remanufactured product, but the pricing and sales are determined by the OEM*.

**Hypothesis** **2.** *The OEM and remanufacturer produce different carbon emissions per unit of new and remanufactured products, thus having different levels of environmental impact. Taking a medium-sized used engine remanufacturing company in China as an example, the carbon emissions per unit of the remanufactured engine are 40% of the carbon emissions per unit of the new engine compared to the new engine. Therefore, the OEM can adjust its own revenue by changing the unit outsourcing cost and new product production to constrain the total carbon emissions and carbon trade volume.*

**Hypothesis** **3.** *Market-based policies of carbon, that is, the carbon-emission-constraint and carbon-trade policies, are the policies adopted by the government to restrain the excessive carbon emission of enterprises and are two important policies to promote the development of the remanufacturing industry. There are numerous policies to promote the development of the outsourcing remanufacturing industry, such as the government carbon tax policy, carbon subsidy policy, carbon regulation policy, and so on. At present, there are few comparative analyses on the impact of various policies on outsourcing remanufacturing, especially a comparative study on the impact of carbon-emission-constraint and carbon-trade policies on outsourcing remanufacturing*.

**Hypothesis** **4.** *By drawing on the literature [14,56], under the carbon-emission-constraint policy, it is stipulated that the total carbon emissions of new and remanufactured products in the carbon market will not exceed T. Under the carbon-trade policy, it is stipulated that the OEM will purchase credits in the carbon market when its carbon emissions are greater than T. The article uses this constraint to analyze the impact of carbon-emission-constraint policy and carbon-trade policy on the output, price, and environmental impact of new and remanufactured products. By setting a cap on carbon emissions, we analyze which is more beneficial to the development of outsourcing remanufacturing: the carbon-emission-constraint policy or the carbon-trade policy*.

**Hypothesis** **5.** *A credit market exists for the OEM under the carbon-trade policy. When the total carbon emissions of both the OEM and the remanufacturer exceed the carbon cap, the OEM and the remanufacturer can offset their carbon emissions even if there are no excess credits in the market. The OEM and the remanufacturer can purchase any number of credits in the credit market without affecting the credit price.*

## 3. Model Analysis

### 3.1. Model Establishment

(1) When the government does not adopt any policy, the income of OEM can be expressed in Equation (1), while the income of the remanufacturer can be expressed in (2):
  (1) $$\pi_{CEn}(q_{CEn},w_{CE})=(p_{CEn}-c_{n})q_{CEn}+(p_{CEr}-w_{CE})q_{CEr}$$
  (2) $$\pi_{CEr}=(w_{CE}-c_{r})q_{CEr}-\frac{k}{2}{\left(q_{CEr}\right)}^{2}$$
(2) When the government adopts the carbon-emission-constraint policy, the income of the OEM can be shown in Equation (3), while the income of the remanufacturer can be shown in (4):
  (3) $$\left\{\begin{array}{l} \max\pi_{COn}(q_{COn},w_{CO})=(p_{COn}-c_{n})q_{COn}+(p_{COr}-w_{CO})q_{COr}\\ s.t.\;e_{n}q_{COn}+e_{r}q_{COr}=T \end{array}\right.\;$$
  (4) $$\pi_{COr}=(w_{CO}-c_{r})q_{COr}-\frac{k}{2}{\left(q_{COr}\right)}^{2}$$
(3) When the government uses the carbon-trade policy, the revenues of the OEM and the remanufacturer are shown in Equations (5) and (6), separately:
  (5) $$\pi_{CDn}=(p_{CDn}-c_{n})q_{CDn}+(p_{CDr}-w_{CD})q_{CDr}-(e_{n}q_{CDn}-T)Q\;$$
  (6) $$\pi_{CDr}=(w_{CD}-c_{r})q_{CDr}-\frac{k}{2}{\left(q_{CDr}\right)}^{2}+(T-e_{r}q_{CDr})Q$$

### 3.2. Model Solution

Lemma 1 is presented to obtain the optimal solution under different policies.

**Lemma** **1.** (i) In Equation (2), $\pi_{CEr}$ is a concave function about $\tau_{CE}$; in Equation (1), $\pi_{CEn}$ is a concave function about $q_{CEn},w_{CE}$. (ii) In Equation (4), $\pi_{COr}$ is a concave function about $\tau_{CO}$; in Equation (1), $\pi_{COn}$ is a concave function about $q_{COn},w_{CO}$. (iii) In Equation (6), $\pi_{CDr}$ is a concave function about $\tau_{CD}$; in Equation (1), $\pi_{CDn}$ is a concave function about $q_{CDn},w_{CD}$.

The proof of Lemma 1 is shown in Appendix A.

The optimal solutions with different policies can be calculated according to Lemma 1:

**Conclusion** **1.** The optimal solutions involve the policies of carbon-emission-constraint and carbon-trade (see Table 2).

The proof of Conclusion 1 is shown in Appendix A. In Table 2, we used Q=0 in the optimal solution of the CD mode to obtain the optimal solution of CE mode, which is not listed here.

### 3.3. Model Analysis

By comparing and analyzing the optimal solution under different modes, this article can obtain the influence of the government’s implementation of different policies on outsourcing remanufacturing, as shown in the following conclusions.

First of all, if the upper limit of carbon emissions exceeds a particular threshold, it does not affect the OEM’s production decisions. Therefore, the following analysis should be performed under the condition of Hypothesis 6:

**Hypothesis** **6.** *The upper limit of carbon emissions should meet*

$$T<\frac{e_{n}}{2}+\frac{(c_{r}-\delta c_{n})e_{r}+\left[(k+\delta )c_{n}-\delta c_{r}\right]e_{n}}{2(\delta^{2}-\delta -k)}.$$

Otherwise, the carbon emission constraint and the carbon trade does not change the production decisions of the two manufacturers.

**Conclusion** **2.** The effect of different kinds of government policies on the unit retail price: For ease of expression, set

$$M=\frac{e_{n}}{2}+\frac{(c_{r}-\delta c_{n})e_{r}+\left[(k+\delta )c_{n}-\delta c_{r}\right]e_{n}}{2(\delta^{2}-\delta -k)}$$

$$N=M+\frac{Q\left[(k+\delta )e_{n}^{2}+e_{r}^{2}-2\delta e_{n}e_{r}\right]}{2(\delta^{2}-\delta -k)}$$

*(i)*  *When*
$$T<N,\;p_{COn}^{*}>p_{CDn}^{*}>p_{CEn}^{*};$$
  *when*
$$N\leq T<M,\;p_{CDn}^{*}\geq p_{COn}^{*}>p_{CEn}^{*}.$$
*(ii)*  *When*
$$T<N,\;p_{COr}^{*}>p_{CDr}^{*}>p_{CEr}^{*};$$
  *when*
$$N\leq T<M,\;p_{CDr}^{*}\geq p_{COr}^{*}>p_{CEr}^{*}.$$

The proof of Conclusion 2 is shown in Appendix A. Similar to [56], Conclusion 2 indicates that both the carbon-emission-constraint and the carbon-trade policy can enhance the price of remanufactured and new products. Additionally, when the upper limit of carbon emissions is lower than a particular threshold, the unit retail price of both products is higher under the carbon-emission-constraint policy. Nevertheless, when the upper limit of carbon emissions is more than this threshold, the unit retail price of both products is higher under the carbon-trade policy.

The two manufacturers’ production is subject to the policies of carbon-emission-constraint and carbon-trade. In the context of the carbon-emission-constraint policy, if the OEM increases the unit outsourcing cost to scale up remanufactured product production [60], the production cost would be indirectly raised. If an OEM increases the output of new products, its production activities would be limited by the higher carbon emissions generated by the new products. Consequently, the OEM compensates for the loss by raising the price of products so that the OEM can maximize its profits. Within the carbon-trade policy, the production cost for new products rises, and OEMs indirectly increase the cost for remanufactured products by raising the unit outsourcing cost. In addition, the OEM also passes on the increased cost to consumers by raising product prices. In contrast to [56], Conclusion 2 further indicates how the upper limit of carbon emissions affects product prices under different policies. When the upper limit of carbon emissions is less than a certain threshold, it limits the production of the two manufacturers significantly; the OEM can produce more new products by buying carbon emissions under the carbon-trade policy. The increase in the production of new products leads to a rise in production costs, which drives up prices; on the flip side, the output drives down the product price. When the upper limit of carbon emissions is relatively low, the impact of the production cost’s increase on price is lower than that of production increase, so the unit retail price under the carbon-emission-constraint policy is higher. When the upper limit of carbon emissions is above that threshold, it causes a little restriction on the production of the two manufacturers [61]. As a result, under the carbon-trade policy, the increase in production cost has a higher impact on the price than the increase in output, so the unit retail price under the carbon-emission-constraint policy is lower.

**Management Enlightenment 1:** The unit retail price of both products varies with different policies set by the government, and the unit retail price under different policies is related to the carbon emissions cap. Both the carbon-emission-constraint and carbon-trade policies can boost the unit retail price. The unit retail price under the carbon-emission-constraint policy is the highest when the upper limit of carbon emissions is below a specific threshold, and the unit retail price under the carbon-trade policy is the highest when the upper limit of carbon emissions is greater than that threshold. The rising price of products increases the purchase cost and burden of consumers, reducing their purchasing enthusiasm. Therefore, when the upper limit of carbon emissions is below a particular threshold, the carbon-trade policy is more friendly to consumers. However, when the upper limit of carbon emissions exceeds this threshold, the government should implement a carbon-emission-constraint policy to benefit consumers.

**Conclusion** **3.** The effect of different policies on the sales volume of both products:

*(i)*  *When*
$$T<N,\;q_{CEn}^{*}>q_{CDn}^{*}>q_{COn}^{*};$$
  *when*
$$N\leq T<M,\;q_{CEn}^{*}>q_{COn}^{*}\geq q_{CDn}^{*}.$$
*(ii)*  *When*
$$\delta <\frac{e_{r}}{e_{n}}\;and\;T<N,\;q_{CEr}^{*}>q_{CDr}^{*}>q_{COr}^{*};$$
  *when*
$$\delta <\frac{e_{r}}{e_{n}}\;and\;N\leq T<M,\;q_{CEr}^{*}>q_{COr}^{*}\geq q_{CDr}^{*}.$$
  *When*
$$\delta \geq \frac{e_{r}}{e_{n}}\;and\;T<N,\;q_{CEr}^{*}\leq q_{CDr}^{*}\leq q_{COr}^{*};$$
  *when*
$$\delta \geq \frac{e_{r}}{e_{n}}\;q_{CEr}^{*}\leq q_{COr}^{*}\leq q_{CDr}^{*}\;and\;N\leq T<M.$$

Similar to [29,56], Conclusion 3 shows that both the carbon-emission-constraint and carbon-trade policies can lead to a decrease in the sales volume of new products. Both government-imposed policies reduce the sales of remanufactured products when the discount rate is lower than the proportion of carbon emissions for both products. Both policies increase sales of remanufactured products if the discount rate is more than the carbon emissions ratio of the two products [62]. In contrast to the results in [29], we discovered that the volume of product sales under both policies is related to the carbon emissions cap and that the discount rate and the ratio of carbon emissions for both products also affect the sales volume for the remanufactured products in the two policies.

The policies of carbon emission constraint and carbon trade implemented by the government limit the carbon emissions of the two manufacturers, then restrict the production of new products with high carbon emissions [63], resulting in the output of new products being lower than that without any government policy. According to Conclusion 2, the OEM will make up for the loss by raising the price of new products. As the price rises, consumers’ purchasing power declines, which makes the sales of new products reduce under the policies of carbon emission constraint and carbon trade. When the cap on carbon emissions is under a particular threshold, the new products under the carbon-emissions-constraint policy have a higher unit retail price than under the carbon-trade policy. In this case, the carbon-emission-constraint policy is more restrictive to consumers’ purchasing power, and therefore more new products are sold under the carbon-trade policy. When the cap on carbon emissions is above this threshold, new products under the carbon-emission-constraint policy have a lower unit retail price than under the carbon-trade policy. Thus, the carbon-trade policy is more restrictive to consumers’ purchasing power, and more new products are sold under the carbon-emission-constraint policy.

There is no significant consumer preference over remanufactured products as the discount rate is lower than the carbon emissions ratio of the two products. When the carbon emissions cap is below a specific threshold, remanufactured products under the carbon-emission-constraint policy have a higher retail price per unit. Consumers decide their purchasing behavior based on the product price, so more remanufactured products are sold under the carbon-trade policy. When the carbon emission cap exceeds this threshold, the price of remanufactured products under the carbon-emission-constraint policy is lower, and more remanufactured products are sold under the carbon-constraint policy at this time. Under the condition that the discount rate is more than the carbon-emissions ratio of the two products, consumers prefer to buy remanufactured products, and the influence of remanufactured products’ price on consumers’ purchasing behavior is weakened at this time. At this point, the OEM takes measures, such as increasing the unit outsourcing cost, to encourage the remanufacturer to expand production. When the upper limit of carbon emissions is less than this threshold, considering the higher price of remanufactured products under the carbon-emission-constraint policy, the OEM stimulates the remanufacturer to manufacture further remanufactured products for more profit. Thus, the sales volume of remanufactured products under the carbon-emission-constraint policy is more than that under the carbon-trade policy. When the upper limit of carbon emissions is greater than this threshold, the remanufactured products under the carbon-trade policy are more expensive; the OEM stimulates the remanufacturer to produce more remanufactured products to maximize profits. Therefore, more remanufactured products are sold under the carbon-trade policy than under the carbon-emission-constraint policy.

**Management Enlightenment 2:** Both the carbon-emission-constraint and carbon-trade policies will influence the sales volume of remanufactured and new products. The volume of product sales under different policies is related to the cap on carbon emissions. In addition, consumers’ preference for remanufactured products can also affect remanufactured products’ sales volume. To promote green and low-carbon production, the government should take measures to enhance consumers’ awareness of green consumption. Consumers’ preference for remanufactured products helps stimulate remanufacturers to expand their production scale, improve production technology, and reduce production costs, thus promoting green and low-carbon production.

**Conclusion** **4.** The effect of different government policies on the unit outsourcing cost:

*For ease of expression, set*

$$X=M+\frac{Q\left[(k+\delta )e_{n}^{2}+e_{r}^{2}-2\delta e_{n}e_{r}\right]\left[k\delta e_{n}+e_{r}(2\delta -2\delta^{2}+k)\right]}{2k(\delta^{2}-\delta -k)(\delta e_{n}-e_{r})}\;$$

*(i)*  *When*
$$\delta \leq \frac{e_{r}}{e_{n}},\;\;w_{CO}^{*}<w_{CE}^{*}<w_{CD}^{*};$$
*(ii)*  *when*
$$\delta >\frac{e_{r}}{e_{n}}\;and\;\;T<X,\;\;w_{CE}^{*}<w_{CD}^{*}<w_{CO}^{*};$$
  *when*
$$\delta >\frac{e_{r}}{e_{n}}\;and\;\;X\leq T<M,\;\;w_{CE}^{*}<w_{CO}^{*}\leq w_{CD}^{*}.$$

Compared to the existing literature, Conclusion 4 shows that different policies implemented by the government can affect the outsourcing cost of remanufactured products. The highest cost per unit of outsourcing under the carbon-trade policy and the lowest cost per unit of outsourcing under the carbon-emission-constraint policy occurs where the discount rate for the two products is lower than the proportion of the two products’ carbon emissions. The unit outsourcing cost is related to the cap on carbon emissions set by the government if the discount rate is higher than the proportion for both products on carbon emissions. The unit outsourcing cost is maximal under the carbon-trade policy when the cap on carbon emissions is below a particular threshold. Additionally, when the cap on carbon emissions is more than that threshold, the unit outsourcing cost is highest under the carbon-emission-constraint policy.

According to Conclusion 3, the volume of sales of remanufactured and new products is below the ordinary volume of sales when the discount rate is lower than the proportion of carbon emissions of the two products, regardless of whether the carbon-emission-constraint policy or the carbon-trade policy implemented by the government. When the government implements the carbon-trade policy, the cost of producing the new product increases, and the OEM increases the unit retail price. As a result, new products become less attractive to consumers, and new products have a lower sales volume. To be more profitable, the OEM has to encourage remanufacturers to produce more remanufactured products by increasing the outsourcing cost per unit. In addition, according to Conclusion 2, with the increase in the unit retail price of both products, remanufacturers want to increase their revenue by asking the OEM to increase the outsourcing cost. Therefore, the unit outsourcing cost under the carbon-trade policy is higher than that under the carbon-emission-constraint policy. When the government implements the carbon-emission-constraint policy, the upper limit of carbon emissions can limit manufacturers’ production behavior, and consumers have no obvious preference for remanufactured products. In this case, remanufactured products are less profitable, and the OEM does not set a high outsourcing cost, so the unit outsourcing cost is lower under the carbon-emission-constraint policy than under the carbon-trade policy.

When the discount rate is above the carbon emissions ratio of the two products, consumers prefer remanufactured products. Compared with the carbon-emission-constraint policy, the OEM can purchase additional carbon emission credits under the carbon-trade policy. In this case, the upper limit of carbon emissions has less constraint on the OEM; the OEM attempts to profit by expanding the market for remanufactured products and selling remanufactured products in large quantities. Therefore, when the discount rate is higher than the proportion of carbon emissions of the two products, the outsourcing cost under the carbon-trade policy is higher than that without any government policy, which is similar to [14]. Additionally, the unit outsourcing cost depends on the upper limit of carbon emissions. According to Conclusion 4, the highest volume of sales of remanufactured products under the carbon-emission-constraint policy occurs when the government sets a low cap on carbon emissions. In this case, the policy has a high production constraint on the OEM, and thus the OEM will raise the outsourcing cost to facilitate the manufacturing and selling of remanufactured products. In short, the outsourcing cost is highest under the carbon-emission-constraint policy when the discount rate is above the proportion of carbon emissions of both products and the cap of carbon emissions is below a certain threshold. However, when the cap on carbon emissions is above this threshold, the carbon-emission-constraint policy is less restrictive for the OEM when the volume of remanufactured products sold under the carbon-trade policy is highest. Therefore, in this case, the unit outsourcing cost is the highest under the carbon-trade policy.

**Management Enlightenment 3:** Both consumers’ appetite for remanufactured products and the cap on carbon emissions can affect the outsourcing decisions of OEM. Therefore, the OEM should take consumers’ preferences into account and make reasonable outsourcing costs to stabilize the market balance between remanufactured and new products. In addition, the government should formulate appropriate low-carbon policies. On the one hand, the reasonable upper limit of carbon emissions could be formulated according to consumers’ preferences, and the interests of all parties in the supply chain should be comprehensively considered. On the other hand, consumers should be actively guided to strengthen the market of remanufactured products.

**Conclusion** **5.** The environmental impact of different government policies:

*(i)*  *The impact of the OEM on the environment when the government implements different policies:*
*When*
$$T<N,\;E_{COn}<E_{CDn}<E_{CEn};$$
  *when*
$$N\leq T<M,\;\;E_{CDn}\leq E_{COn}<E_{CEn}.$$
*(ii)*  *The impact of the remanufacturer on the environment when the government implements different policies:*
*When*
$$\delta <\frac{e_{r}}{e_{n}}\;and\;T<N,\;\;E_{COr}<E_{CDr}<E_{CEr};$$
  *when*
$$\delta <\frac{e_{r}}{e_{n}}\;and\;N\leq T<M,\;\;E_{CDr}\leq E_{COr}<E_{CEr};$$
  *when*
$$\delta \geq \frac{e_{r}}{e_{n}}\;and\;T<N,\;\;E_{CEr}<E_{CDr}<E_{COr};$$
  *when*
$$\delta \geq \frac{e_{r}}{e_{n}}\;and\;N\leq T<M,\;\;E_{CEr}<E_{COr}\leq E_{CDr}.$$
*(iii)*  *The total impact of the OEM and the remanufacturer on the environment when the government implements different policies:*
*Set*
$$Z=e_{n}\left[\delta -\delta^{2}+k-(\delta +k)(c_{n}+e_{n}Q)+\delta (c_{r}+e_{r}Q)\right]+e_{r}\left[\delta (c_{n}+e_{n}Q)-(c_{r}+e_{r}Q)\right]\;$$
  *When*
$$T<\frac{Z}{2(\delta -\delta^{2}+k)},\;\;E_{CO}<E_{CD}<E_{CE};$$
  *when*
$$\frac{Z}{2(\delta -\delta^{2}+k)}\leq T<M,\;\;E_{CD}\leq E_{CO}<E_{CE}.$$

According to Conclusion 3, when the carbon emissions cap is low, new products are sold in the highest volume without any government policy, the second-highest volume under the carbon-trade policy, and the lowest volume under the carbon-emission-constraint policy. Therefore, when the cap on carbon emissions is below a particular threshold, the OEM has the minimum environmental impact under the carbon-emission-constraint policy and the maximum environmental impact under no government policy. Additionally, when the cap on carbon emissions is above this threshold, new products’ sales are highest in the absence of any policy and lowest under the carbon-trade policy. Therefore, when the cap on carbon emissions is above a particular threshold, the environmental impact of the new products is the smallest under the carbon-trade policy and the largest without any government policy.

For remanufacturers, in the case that consumers have no apparent preference for remanufactured products, if the carbon emissions cap is below a particular threshold, remanufactured products are sold at the highest level without any policy and at the lowest level with the carbon-emissions-constraint policy. When the carbon emissions cap is above this threshold, remanufactured product sales remain highest without any government policy but lowest under the carbon-trade policy. As a result, when the upper limit of carbon emissions set by the government is lower, the environmental impact of remanufacturers is minimal under the carbon-emission-constraint policy, and when there are no policies, the effect is maximal. When the government sets a high cap on carbon emissions, the environmental impact of remanufacturers is minimal under the carbon-trade policy and maximal without any government policy. In the case where consumers favor remanufactured products when the carbon cap is below a particular threshold, remanufactured products sales are highest under the carbon-emission-constraint policy, second-highest under the carbon-trade policy, and lowest in the absence of any policy. When the cap on carbon emissions is above this threshold, remanufactured products’ sales are highest under the carbon-trade policy, second-highest under the carbon-constraint policy, and lowest under no policy at all. In other words, when the upper limit set by the government is lower, the impact of the remanufacturers on the environment is maximal under the carbon-emission-constraint policy, and it is minimal when the government does not implement any policy. When a higher cap on carbon emissions is set, the environmental impact of remanufacturers is maximal under the carbon-trade policy and minimal under no policy at all.

In terms of the overall environmental impact of both manufacturers, similar to [56], the overall environmental impact is the greatest when the government does not implement any policies. In contrast to [56], we argue that when the upper limit of carbon emissions is lower than a particular threshold, the overall environmental impact of the supply chain under the carbon-trade policy is higher than that under the carbon-emission-constraint policy; the aggregate environmental impact of the carbon-emission-constraint policy is greater than the aggregate environmental impact of the carbon-trade policy. In summary, the environmental impact of manufacturers is largely dependent on the government-mandated cap on carbon emissions. The OEM makes production decisions based on government policies to reduce the impact on the environment. In addition, all parties in the supply chain should actively improve low-carbon technology to decrease carbon emissions. The government should formulate reasonable policies on carbon emissions to guide enterprises’ production and meet the target of low carbon emissions.

## 4. Numerical Analysis

For additional verification of the conclusions and discussion of the impact of carbon-emission-constraint and carbon-trade policies on outsourcing remanufacturing, this article takes a mid-sized used engine remanufacturing company in China as an example for simulation analysis. According to [64], we take $e_{n}=1$, $e_{r}=0.4$, $c_{n}=0.2$, $c_{r}=0.1$, k=1.1, δ=0.6. According to Hypothesis 6:

$$T<\frac{e_{n}}{2}+\frac{(c_{r}-\delta c_{n})e_{r}+\left[(k+\delta )c_{n}-\delta c_{r}\right]e_{n}}{2(\delta^{2}-\delta -k)}.$$

We know that $T\in \left[0,0.2\right]$ satisfies the hypothesis in this paper. Meanwhile, it can be determined that $\delta >\frac{e_{r}}{e_{n}}$, which means that consumers prefer remanufactured products.

### 4.1. The Influence of Carbon Emission Constraint and Carbon Trade on the Unit Selling Price

**Figure 1:**
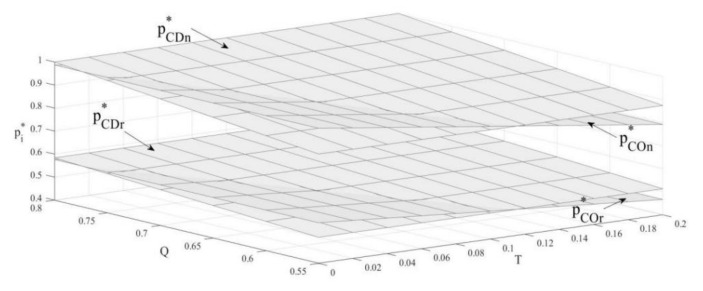
The influence of carbon emission constraint and carbon trade on the unit selling price.

According to Figure 1, when the upper limit of carbon emissions is lower than a certain threshold, remanufactured and new products’ unit retail price is higher under the carbon-emission-constraint policy, while lower under the carbon-trade policy. Additionally, when the upper limit of carbon emissions is higher than the threshold, the two products’ unit retail price is higher under the carbon-trade policy, while it is lower under the carbon-emission-constraint policy. The reason for this is that when the emissions cap is lower, the carbon-emission-constraint policy greatly restricts the production of the OEMs, causing them to reduce production and increase prices. Therefore, in this case, both products have a higher retail price per unit under the carbon-emission-constraint policy. However, when the carbon emissions cap is higher, the policy of carbon emissions constraint reduces production constraint on the OEM, while the carbon-trade policy prompts the OEM to engage in carbon trading, which increases their cost. Thus, in this case, both products have a higher unit retail price under the carbon-trade policy. In contrast to [29,56], we can find that the policy does not affect the relative price of remanufactured and new products because the cost of producing a new product is always higher than the cost of a remanufactured product. That is to say, new products always retail at a higher price than remanufactured products.

**Corollary** **1.** The influence of a carbon emissions cap on the unit retail price of two products under the carbon-emission-constraint policy:

$$\frac{\partial p_{COn}^{*}}{\partial T}<0;\;\frac{\partial p_{COr}^{*}}{\partial T}<0$$

**Corollary** **2.** The influence of carbon-trade price on the unit retail price of two products under the carbon-trade policy:

$$\frac{\partial p_{CDn}^{*}}{\partial Q}>0;\;\frac{\partial p_{CDr}^{*}}{\partial Q}>0$$

Similar to [56], Corollary 1 and Corollary 2 show that the retail price per unit of remanufactured and new products decreases with the increase in the carbon emissions cap under the carbon-emission-constraint policy, while the retail price per unit of these two products increases with the carbon-trade price under the carbon-trade policy. Under the carbon-emission-constraint policy, the increase in the carbon cap reduces the restrictions on OEM production. Thus, their production increases, and the unit retail price decreases accordingly. However, under the carbon-trade policy, the rise of carbon-trade price means due to the increase in production cost, OEMs increase the unit retail price to maintain revenue.

### 4.2. The Influence of Carbon Emission Constraint and Carbon Trade on Sales Volume

**Figure 2:**
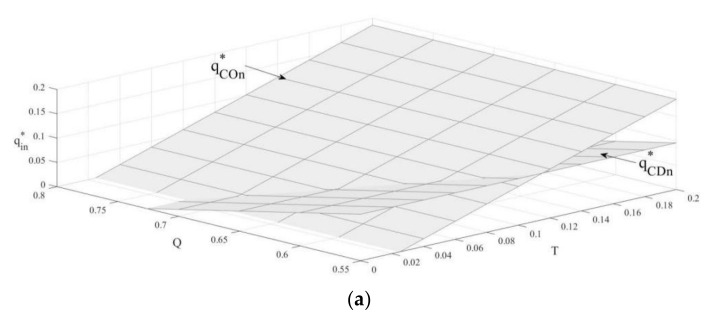

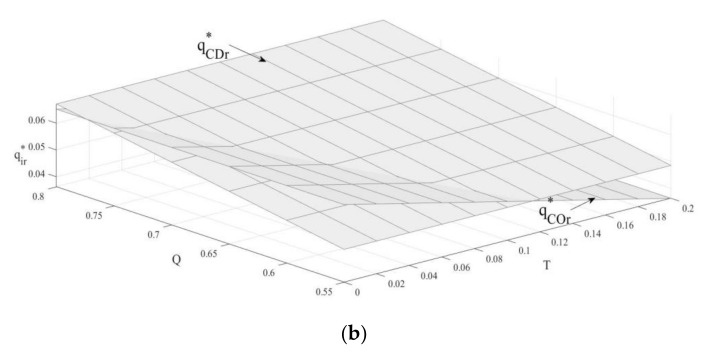
The influence of carbon emission constraint and carbon trade on sales volume. (**a**) The influence on new products. (**b**) The influence on remanufactured products.

According to Figure 2, when the cap on carbon emissions falls below a particular threshold, new products’ sales volume is greater under the carbon-trade policy, and the sales volume of remanufactured products is greater under the carbon-emission-constraint policy. When the upper limit of carbon emissions is higher than this threshold, the sales volume of new products is greater under the carbon-emission-constraint policy, and the sales volume of remanufactured products is greater under the carbon-trade policy. Therefore, to facilitate the growth of remanufacturing, the government should set a higher carbon emissions cap and implement it in combination with the carbon-trade policy, or implement the carbon-emission-constraint policy and set a lower carbon emissions cap. Meanwhile, the government should vigorously promote low-carbon environmental protection and enhance consumers’ preference for remanufactured products.

**Corollary** **3.** The influence of a carbon emissions cap on the sales volume of two products under the carbon-emission-constraint policy:

$$\frac{\partial q_{COn}^{*}}{\partial T}>0;\;\frac{\partial q_{COr}^{*}}{\partial T}<0$$

**Corollary** **4.** The influence of carbon-trade price on the sales volume of two products under the carbon-trade policy:

$$\frac{\partial q_{CDn}^{*}}{\partial Q}<0;\;\frac{\partial q_{CDr}^{*}}{\partial Q}>0.$$

In contrast to [29], Corollary 3 and Corollary 4 show that under the carbon-emission-constraint policy, as the carbon emissions cap increases, the sales volume of new products increases while the sales volume of remanufactured products decreases. However, under the carbon-trade policy, sales of new products fall as the price of carbon trade rises, while sales of remanufactured products rise.

By combining Corollary 1 and Corollary 2, under the carbon-emission-constraint policy, the increase in the upper limit of carbon emissions lowers new products’ unit selling price and raises the volume of new products sold. Since new products are more expensive than remanufactured products, OEMs aggressively sell new products and reduce remanufactured product sales to increase their revenue. Under the carbon-trade policy, the increase in the price of carbon trade increases the cost of the production of new products, leading to a decrease in their sales volume. When consumers prefer remanufactured products, they choose them to replace the consumption of new products, namely, the remanufactured products’ sales increase.

### 4.3. The Influence of Carbon Emission Constraint and Carbon Trade Policies on the Unit Outsourcing Cost

**Figure 3:**
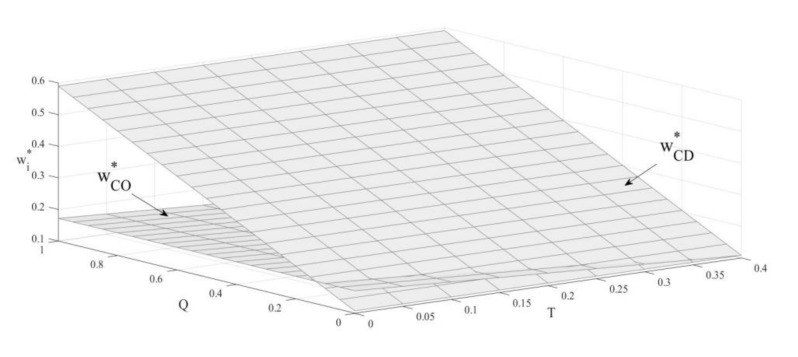
The influence of carbon emission constraint and carbon trade on the unit outsourcing cost.

According to Figure 3, when the upper limit of carbon emissions is below a particular threshold, the unit outsourcing cost of the product under the carbon-emission-constraint policy is higher than the cost under the carbon-trade policy. When the cap on carbon emissions is higher than the threshold, the outsourcing cost under the carbon-trade policy is higher. When the cap on carbon emissions is lower, the OEM has to reduce production and increase the outsourcing cost to encourage remanufacturers. Therefore, when the cap on carbon emissions is lower, the outsourcing cost under the carbon-emission-constraint policy is higher. When the carbon emission cap is high, the unit price and the volume of remanufactured products sold under the carbon-trade policy are higher due to the clear consumer appetite for remanufactured products [60]. Additionally, remanufacturers demand increased outsourcing costs to increase their income. Therefore, when the government sets a higher carbon emission cap, the outsourcing cost under the carbon-trade policy is higher.

**Corollary** **5.** The influence of a carbon emissions cap on the unit outsourcing cost under the carbon-emission-constraint policy:

$$\frac{\partial w_{CO}^{*}}{\partial T}<0.$$

**Corollary** **6.** The influence of carbon trading price on the unit outsourcing cost under the carbon-trade policy:

$$\frac{\partial w_{CD}^{*}}{\partial Q}>0.$$

Similar to [14], Corollary 5 and Corollary 6 show that the unit outsourcing cost decreases as the carbon emissions cap increases in the case of the carbon-emission-constraint policy, while in the case of the carbon-trade policy, the unit outsourcing cost goes up with the rise of the carbon-trade price. With the carbon-emission-constraint policy, the rise in the carbon emissions cap means that the OEM can produce more products, thus reducing the cost of outsourcing. Under the carbon-trade policy, the rise of the carbon-trade price increases the OEM’s production cost, and the demand for remanufactured products increases accordingly, prompting the OEM to increase the outsourcing cost. The government can choose the carbon-trade policy and formulate a reasonable carbon-trade price to facilitate the development of remanufacturing, influencing the decisions of two manufacturers by adjusting policies to enhance the revenue of the remanufacturer and promote low-carbon development.

In this section, an example of a medium-sized used engine remanufacturing enterprise in China is taken for simulation analysis. By assigning values to the carbon emissions of two products, production costs per product, recycling coefficients of used products, and consumer preferences for remanufactured products in the model constructed in the article, the impact of the carbon-emission-constraint and carbon-trade policies on the retail price per unit of product, sales volume, and outsourcing cost per unit of product is shown in the form of images. The results of the simulation analysis in this study show that the unit retail price, sales volume, and outsourcing cost per unit of product are not higher under a certain policy but depend on the range of the carbon emission cap. The results of the analysis in this section not only verify the above conclusions but also more intuitively and graphically represent the impact of the two policies on outsourcing remanufacturing production.

## 5. Conclusions and Discussion

### 5.1. Conclusions

Based on the outsourcing remanufacturing model, a game model between the OEM and the remanufacturers is developed in this article, aiming to investigate the impact of the carbon-emission-constraint and carbon-trade policies on the model. By comparing and analyzing the impact of different policies on the unit retail price, the volume of sales, and the environmental impact of remanufactured and new products, this article draws the following conclusions:

(1) In the closed-loop supply chain that consists of the OEM and the remanufacturer, the implementation of the carbon-emission-constraint policy and carbon-trade policy by the government leads to an increase in the unit retail price of both remanufactured and new products, which is similar to [56]. Different from [56], this study further indicates how the cap on carbon emissions affects product prices under different policies. When the cap on carbon emissions is below a particular threshold, the retail price per unit of product is higher under the carbon-emission-constraint policy; otherwise, it is higher under the carbon-trade policy;
(2) Similar to the literature [29,56], both policies result in a decrease in new products sales, but remanufactured products sales only decrease if the discount rate is lower than the proportional carbon emissions of the two products; otherwise, remanufactured products’ sales increase. In addition, new products sales are correlated with the upper limit of carbon emissions under the two policies. If the cap on carbon emissions is below a particular threshold, more new products are sold under the carbon-trade policy; otherwise, more are sold under the carbon-emission-constraint policy. However, compared with the results of the literature [29], the volume of remanufactured products sold under both policies is not only related to the carbon emissions cap but is also influenced by the ratio of carbon emissions and the discount rate;
(3) In comparison with the existing literature, the OEM will change remanufactured products’ unit outsourcing costs as the government adopts different policies. Additionally, if the discount rate is above the carbon emissions ratio of both products and higher than a specific threshold, or if the discount rate is below the carbon emissions ratio of both products, the unit outsourcing cost would be higher under the carbon trading policy. At the same time, the outsourcing cost would be higher under the carbon-emission-constraint policy in other cases;
(4) The two policies implemented by the government can reduce the environmental impact of manufacturing production as a whole. Compared to [14,56], this study also compared the magnitude of the environmental impact of the two policies. When the upper limit of carbon emissions is below a particular threshold, the influence of a carbon-emission-constraint policy on the environment is lower; otherwise, the environmental impact of a carbon-trade policy is lower. However, combined with Conclusion 2 and the positive correlation between sales volume and carbon emissions, it can be seen that remanufactured and new products are not always environmentally friendly under the carbon-emission-constraint policy and carbon-trade policy. This also suggests that when consumer environmental awareness is higher, the two policies effectively facilitate the production of remanufactured products and reduce the comprehensive influence of the two manufacturers’ production on the environment in the supply chain.

### 5.2. Discussion

This article comparatively investigates the effect of carbon-emission-constraint and carbon-trade policies on the production behavior of both manufacturers, which differs from previous studies on the impact of carbon-reduction policies on supply-chain-reduction models, corporate financing decisions, and product pricing. The analysis of this article is limited to the outsourcing remanufacturing model, in which the two carbon-reduction policies are analyzed in comparison. Therefore, this article provides a foundation for the government to select appropriate policies, setting a suitable carbon emissions cap and carbon trading price in practice to promote the coordination between the OEM and remanufacturer in the closed-loop supply chain and effectively realize low-carbon production. In addition, it can be concluded from the analysis results that consumers’ environmental awareness plays a significant role in the production decisions of manufacturers. Additionally, the government ought to undertake active steps to encourage manufacturers to achieve green and low-carbon production, such as widely carrying out low-carbon publicity, advocating consumers to carry out green consumption, and enhancing consumers’ environmental awareness.

However, this study also has some limitations. The conclusions derived from the model on the impact of policies on outsourced remanufacturing only hold if the assumptions are met, so the reference effect on the government’s implementation of the carbon-constraint and carbon-trading policies is only applicable under limited circumstances, and future studies can improve the hypothetical conditions to make them more relevant to realistic scenarios. Based on the above findings, this study could be scaled up in three areas for the future. Firstly, the outsourcing remanufacturing model can be extended to a multi-period model for further exploration. Furthermore, we could add the carbon tax policy into the model and analyze the influence of different government policies on the outsourcing remanufacturing industry. Lastly, it is necessary to comprehensively explore the influence of government policies on the outsourcing remanufacturing mode, authorization remanufacturing mode, and other remanufacturing modes as China defines the carbon peak and carbon-neutral goals.

## Figures and Tables

**Table 1:** Definition of symbols.

Symbol	Definition
CE	The government is not taking any policy;
CO	The government takes the carbon-emission-constraint policy;
CD	The government takes the carbon-trade policy;
n,r	OEM, remanufacturer;
$q_{in}$ $,q_{ir}$	Number of sales for two kinds of products when the government takes policy i;
$p_{in}$ $,p_{ir}$	The unit retail price for two kinds of products when the government takes policy i;
$c_{n}$ $,c_{r}$	Unit production cost for two kinds of products (from the real-world situation, it is known that $\;c_{n}>c_{r}$);
T	The government sets a carbon-emission limit for OEMs; (namely the carbon emissions cap);
Q	Unit of carbon emissions’ trade price;
$e_{n}$ $,e_{r}$	Carbon emissions per unit of the two kinds of products, also known as the environmental impact per unit of the two kinds of products (from the real-world situation, it is known that $\;e_{n}>e_{r}$);
$E_{in}$ $,E_{ir}$	The total carbon emissions of two kinds of products when the government takes policy i, that is $E_{in}=e_{n}q_{in}$, $E_{ir}=e_{r}q_{ir}$;
$E_{i}$	The total carbon emissions of two manufacturers when the government takes policy i, it is also known as the total environmental influence of the production for both manufacturers.
$\tau_{i}$	The ratio of the volume of used products recycled by remanufacturers to the volume of new products sold, also known as the used product recycling rate (from the real-world situation, it is known that $\;q_{ir}=\tau_{i}q_{in}$);
δ	The ratio of the retail price for each unit of remanufactured product to the retail price for each unit of the new product, which is the consumer preference for remanufactured products (from the real-world situation, it is known that 0≤δ≤1);
$w_{i}$	The outsourcing cost paid by the OEM to the remanufacturer when the production unit remanufactures the product;
$\pi_{in}$ $,\pi_{ir}$	When the government implements policy i, the revenue that the OEM and the remanufacturer receive.

**Table 2:** The optimal solutions under both policies.

Symbol	CO	CD
$q_{in}^{*}$	$\frac{2\delta Te_{r}-2(k+\delta )Te_{n}+\delta e_{n}e_{r}-c_{r}e_{n}e_{r}-e_{r}^{2}+c_{n}e_{r}^{2}}{2(2\delta e_{n}e_{r}-ke_{n}^{2}-\delta e_{n}^{2}-e_{r}^{2})}$	$\frac{\delta -\delta^{2}+k-(\delta +k)(c_{n}+e_{n}Q)+\delta (c_{r}+e_{r}Q)}{2(\delta -\delta^{2}+k)}$
$q_{ir}^{*}$	$\frac{2\delta Te_{n}-2Te_{r}+e_{n}e_{r}-c_{n}e_{n}e_{r}-\delta e_{n}^{2}+c_{r}e_{n}^{2}}{2(2\delta e_{n}e_{r}-ke_{n}^{2}-\delta e_{n}^{2}-e_{r}^{2})}$	$\frac{\delta (c_{n}+e_{n}Q)-(c_{r}+e_{r}Q)}{2(\delta -\delta^{2}+k)}$
$\tau_{i}^{*}$	$\frac{2\delta Te_{n}-2Te_{r}+e_{n}e_{r}-c_{n}e_{n}e_{r}-\delta e_{n}^{2}+c_{r}e_{n}^{2}}{2\delta Te_{r}-2(k+\delta )Te_{n}+\delta e_{n}e_{r}-c_{r}e_{n}e_{r}-e_{r}^{2}+c_{n}e_{r}^{2}}$	$\frac{\delta (c_{n}+e_{n}Q)-(c_{r}+e_{r}Q)}{\delta -\delta^{2}+k+\delta (c_{r}+e_{r}Q)-(\delta +k)(c_{n}+e_{n}Q)}$
$w_{CO}^{*}$	$\frac{2k\delta Τe_{n}-2kTe_{r}+ke_{n}e_{r}+4\delta c_{r}e_{n}e_{r}-kc_{n}e_{n}e_{r}-k\delta e_{n}^{2}-2c_{r}e_{r}^{2}-(k+2\delta )c_{r}e_{n}^{2}}{2(2\delta e_{n}e_{r}-ke_{n}^{2}-\delta e_{n}^{2}-e_{r}^{2})}$	
$w_{CD}^{*}$	$\frac{k\delta (c_{n}+e_{n}Q)+(2\delta -2\delta^{2}+k)(c_{r}+e_{r}Q)}{2(\delta -\delta^{2}+k)}$	
$p_{COn}^{*}$	$\frac{(2\delta +\delta c_{n}+c_{r})e_{n}e_{r}+(-2k-2\delta +\delta^{2}-\delta c_{r})e_{n}^{2}+2(k+\delta -\delta^{2})Te_{n}-(1+c_{n})e_{r}^{2}}{2(2\delta e_{n}e_{r}-ke_{n}^{2}-\delta e_{n}^{2}-e_{r}^{2})}$	
$p_{CDn}^{*}$	$\frac{1+c_{n}+e_{n}Q}{2}$	
$p_{COr}^{*}$	$\frac{3\delta^{2}e_{n}e_{r}+(c_{n}+c_{r}-1)\delta e_{n}e_{r}-(\delta +2k+c_{r})\delta e_{n}^{2}-(1+c_{n})\delta e_{r}^{2}+2(1-\delta )\delta Te_{r}+2k\delta Te_{n}}{2(2\delta e_{n}e_{r}-ke_{n}^{2}-\delta e_{n}^{2}-e_{r}^{2})}$	
$p_{CDr}^{*}$	$\delta \frac{\delta -\delta^{2}+k+k(c_{n}+e_{n}Q)+(1-\delta )(c_{r}+e_{r}Q)}{2(\delta -\delta^{2}+k)}$	
$\pi_{CDn}^{*}$	$\frac{{\left[1-(c_{n}+e_{n}Q)\right]}^{2}}{4}+\frac{{\left[\delta (c_{n}+e_{n}Q)-(c_{r}+e_{r}Q)\right]}^{2}}{4(\delta -\delta^{2}+k)}+TQ$	
$\pi_{CDr}^{*}$	$\frac{k}{8}{\left[\frac{\delta (c_{n}+e_{n}Q)-(c_{r}+e_{r}Q)}{\left(\delta -\delta^{2}+k\right)}\right]}^{2}+TQ$	

## Data Availability

The data were taken from a medium-sized used engine remanufacturing firm in China, the Jinan Fuqiang Company.

## Appendix A

**Proof.** Proof of Lemma 1. Taking $q_{COr}=\tau_{CO}q_{COn}$ into Equation (4), we can obtain:

$$\pi_{COr}=(w_{CO}-c_{r})\tau_{CO}q_{COn}-\frac{k}{2}{\left(\tau_{CO}q_{COn}\right)}^{2}$$

The first-order partial derivative and the second-order partial derivative of $\tau_{CO}$ of Equation (4) are as follows:

$$\frac{\partial \pi_{COr}}{\partial \tau_{CO}}=(w_{CO}-c_{r})q_{COn}-k\tau_{CO}q_{COn}^{2}$$

$$\frac{\partial^{2}\pi_{COr}}{\partial \tau_{CO}^{2}}=-kq_{COn}^{2}$$

It can be shown that Equation (4) is a concave function on $\tau_{CO}$; similarly, it can be obtained that Equations (2) and (6) are concave functions on $\tau_{CE}$ and $\tau_{CD}$. Let the first derivative be equal to 0; we can obtain: $\tau_{CO}^{*}=\frac{w_{CO}-c_{r}}{kq_{COn}}$; similarly, we can obtain $\tau_{CE}^{*}$ and $\tau_{CD}^{*}$. Therefore, the remanufacturer’s waste product recovery rate and profit could be derived by solving for the maximum value of the OEM’s objective function. Taking $p_{COn}=1-q_{COn}-\delta q_{COr}$, $p_{COr}=\delta (1-q_{COn}-q_{COr})$, $q_{COr}=\tau_{CO}^{*}q_{COn}$ into Equation (3),

$$\pi_{COn}(q_{COn},w_{CO})=(1-q_{COn}-\delta \frac{w_{CO}-c_{r}}{k}-c_{n})q_{COn}+\left[\delta (1-q_{COn}-\frac{w_{CO}-c_{r}}{k})-w_{CO}\right]\frac{w_{CO}-c_{r}}{k}$$

The Hessian matrix can be gained from the objective profit function of the OEM $H=\left[\begin{array}{cc} -2 & -\frac{2\delta }{k}\\ -\frac{2\delta }{k} & -\frac{2}{k}-\frac{2\delta }{k^{2}} \end{array}\right]$, $|H|=\frac{4}{k^{2}}\left[k+\delta (1-\delta )\right]>0$, and −2<0; therefore, the objective profit function of the OEM is a concave function with respect to $q_{COn}$, $w_{CO}$, and there is an optimal solution. Similarly, it can be obtained that $\pi_{CEn}$ is a concave function about $q_{CEn},w_{CE}$; $\pi_{CDn}$ is a concave function about $q_{CDn},w_{CD}$. The proof of Lemma 1 is completed. □

**Proof.** Proof of Conclusion 1. Based on the theory of nonlinear programming, this article describes the generalized Lagrangian factor λ; according to Equation (3), the OEM’s revenue function is as follows:

$$L=(1-q_{COn}-\delta \frac{w_{CO}-c_{r}}{k}-c_{n})q_{COn}+\left[\delta (1-q_{COn}-\frac{w_{CO}-c_{r}}{k})-w_{CO}\right]\frac{w_{CO}-c_{r}}{k}+\lambda (T-e_{n}q_{COn}-e_{r}\frac{w_{CO}-c_{r}}{k})$$

Set the K-T point to $q_{COn}^{*},w_{CO}^{*}$; then, the K-T condition is as follows:

$$\left\{\begin{array}{l} 1-2q_{COn}-c_{n}-\frac{2\delta (w_{CO}-c_{r})}{k}-\lambda e_{n}=0\\ \frac{-2\delta q_{COn}+\delta -2w_{CO}+c_{r}}{k}-\frac{2\delta (w_{CO}-c_{r})}{k^{2}}-\frac{\lambda e_{r}}{k}=0\\ \lambda \left[T-e_{n}q_{COn}-e_{r}\frac{\left(w_{CO}-c_{r}\right)}{k}\right]=0 \end{array}\right.$$

From the above equation, we can obtain the optimal solution of $q_{COn}^{*},w_{CO}^{*}$; substituting the optimal solution of $q_{COn}^{*}$, $w_{CO}^{*}$ into the functional relation, we can obtain the optimal solution of $\tau_{CO}^{*},q_{COr}^{*},p_{COn}^{*},p_{COr}^{*}$. The first-order partial derivatives of $q_{CDn},w_{CD}$ of Equation (5) are as follows:

$$\frac{\partial \pi_{CDn}}{\partial q_{CDn}}=1-2q_{CDn}-\delta \frac{w_{CD}-c_{r}-e_{r}Q}{k}-c_{n}-\delta \frac{w_{CD}-c_{r}-e_{r}Q}{k}-e_{n}Q\;\frac{\partial \pi_{CDn}}{\partial w_{CD}}=\frac{-\delta q_{CDn}+\delta (1-q_{CDn})-2w_{CD}+c_{r}+e_{r}Q}{k}-\frac{2\delta (w_{CD}-c_{r}-e_{r}Q)}{k^{2}}$$

Let the first derivative be equal to 0; we can obtain the optimal solution of $q_{CDn}^{*},w_{CD}^{*}$; substituting the optimal solution of $q_{CDn}^{*}$, $w_{CD}^{*}$ into the functional relation, we can obtain the optimal solution of $\tau_{CD}^{*},q_{CDr}^{*},p_{CDn}^{*},p_{CDr}^{*},\pi_{CDn}^{*},\pi_{CDr}^{*}$. The proof of Conclusion 1 is completed. □

**Proof.** Proof of Conclusion 2. According to Conclusion 1:

$$p_{COn}^{*}=\frac{(2\delta +\delta c_{n}+c_{r})e_{n}e_{r}+(-2k-2\delta +\delta^{2}-\delta c_{r})e_{n}^{2}+2(k+\delta -\delta^{2})Te_{n}-(1+c_{n})e_{r}^{2}}{2(2\delta e_{n}e_{r}-ke_{n}^{2}-\delta e_{n}^{2}-e_{r}^{2})}$$

$$p_{CDn}^{*}=\frac{1+c_{n}+e_{n}Q}{2}$$

$$p_{CEn}^{*}=\frac{1+c_{n}}{2}$$

Thus,

$$p_{CDn}^{*}-p_{CEn}^{*}=\frac{1+c_{n}+e_{n}Q}{2}-\frac{1+c_{n}}{2}=\frac{e_{n}Q}{2}>0,$$

namely,

$$p_{CDn}^{*}>p_{CEn}^{*}$$

$$p_{COn}^{*}-p_{CEn}^{*}=\frac{c_{r}e_{n}e_{r}-\delta c_{n}e_{n}e_{r}-\delta c_{r}e_{n}^{2}+(k+\delta )c_{n}e_{n}^{2}+(\delta^{2}-\delta -k)e_{n}^{2}-2(\delta^{2}-\delta -k)Te_{n}}{2(2\delta e_{n}e_{r}-ke_{n}^{2}-\delta e_{n}^{2}-e_{r}^{2})}$$

When

$$T<\frac{e_{n}}{2}+\frac{(c_{r}-\delta c_{n})e_{r}+\left[(k+\delta )c_{n}-\delta c_{r}\right]e_{n}}{2(\delta^{2}-\delta -k)}$$

$$p_{COn}^{*}-p_{CEn}^{*}>0$$

Namely,

$$p_{COn}^{*}>p_{CEn}^{*}$$

Set

$$A=c_{r}-\delta c_{n}-2\delta e_{n}Q,\;B=kc_{n}+\delta c_{n}-k-\delta +\delta^{2}-\delta c_{r}$$

$$p_{COn}^{*}-p_{CDn}^{*}=\frac{Ae_{n}e_{r}+Be_{n}^{2}+2(k+\delta -\delta^{2})Te_{n}+(k+\delta )Qe_{n}^{3}+Qe_{n}e_{r}^{2}}{2(2\delta e_{n}e_{r}-ke_{n}^{2}-\delta e_{n}^{2}-e_{r}^{2})}$$

When

$$T<\frac{e_{n}}{2}+\frac{(c_{r}-\delta c_{n})e_{r}+\left[(k+\delta )c_{n}-\delta c_{r}\right]e_{n}+Q\left[(k+\delta )e_{n}^{2}+e_{r}^{2}-2\delta e_{n}e_{r}\right]}{2(\delta^{2}-\delta -k)}=N$$

$$p_{COn}^{*}-p_{CDn}^{*}>0.$$

When

$$T<N,\;p_{COn}^{*}>p_{CDn}^{*}>p_{CEn}^{*};$$

when

$$N\leq T<M,\;p_{CDn}^{*}\geq p_{COn}^{*}>p_{CEn}^{*}.$$

(i) is thus proven. (ii) can be proven similarly. The proof of Conclusion 2 is completed. □
